# Current Status, Diagnosis, and Treatment Recommendation for Tic Disorders in China

**DOI:** 10.3389/fpsyt.2020.00774

**Published:** 2020-08-13

**Authors:** Zhi-Sheng Liu, Yong-Hua Cui, Dan Sun, Qing Lu, Yu-Wu Jiang, Li Jiang, Jia-Qin Wang, Rong Luo, Fang Fang, Shui-Zhen Zhou, Yi Wang, Fang-Cheng Cai, Qing Lin, Lan Xiong, Yi Zheng, Jiong Qin

**Affiliations:** ^1^ Department of Neurology, Wuhan Children’s Hospital, Tongji Medical College, Huazhong University of Science and Technology, Wuhan, China; ^2^ Department of Neurology and Psychiatry, Beijing Children’s Hospital, Capital Medical University, Beijing, China; ^3^ Department of Pediatrics, Peking University First Hospital, Beijing, China; ^4^ Department of Neurology, Children’s Hospital of Chongqing Medical University, Chongqing, China; ^5^ Department of Pediatrics, Third Affiliated Hospital of Xinxiang Medical College, Xinxiang, China; ^6^ Department of Pediatrics, Huaxi Second Hospital of Sichuan University, Chengdu, China; ^7^ Department of Neurology, Pediatric Hospital Affiliated to Fudan University, Shanghai, China; ^8^ Montreal Neurological Institute and Hospital, McGill University, Montreal, QC, Canada; ^9^ Department of Pediatrics, Beijing Anding Hospital, Capital Medical University, Beijing, China; ^10^ Department of Pediatrics, Peking University People’s Hospital, Beijing, China

**Keywords:** tic disorders, Tourette syndrome, diagnosis, pharmacological treatment, behavioral therapy, comorbidity, expert consensus, Chinese population

## Abstract

Tic disorders (TD) are a group neuropsychiatric disorders with childhood onset characterized by tics, i.e. repetitive, sudden, and involuntary movements or vocalizations; and Tourette syndrome (TS) is the most severe form of TD. Their clinical manifestations are diverse; and are often associated with various psychopathological and/or behavioral comorbidities, including attention deficit hyperactivity disorder (ADHD), obsessive-compulsive disorder (OCD), anxiety, depression, and sleep disorders. Individual severity and response to treatment are highly variable, and there are some refractory cases, which are less responsive to conventional TD treatment. TD/TS are also common in the Chinese pediatric population. To help improve the understanding of TD for pediatricians and other health professionals, and to improve its diagnosis and treatment in China, the Chinese Child Neurology Society (CCNS) has developed an *Expert Consensus on Diagnosis and Treatment of TD in China*, which is based on our clinical experience and the availability therapeutic avenues. It is focused on clinical diagnosis and evaluation of TD and its comorbidities, psychological and educational intervention, nonpharmacological therapy, pharmacological treatment, including traditional Chinese medicine and acupuncture, as well as prognosis in children with TD in China. A summary of the current status of TD and up-to-date diagnosis and treatment recommendations for TD in China is presented here.

## Introduction

Tic disorders (TD) are a group of common neuropsychiatric disorders with onset in childhood and adolescence, characterized by sudden, rapid, recurrent, nonrhythmic movements or vocalizations, including some simple forms, such as eye blinking, facial grimacing, and throat clearing; and some complex forms, such as body twisting, coprolalia (uttering socially inappropriate words, such as swearing) or echolalia (repeating the words or phrases of others) (1–3). TD is often associated with various psychopathological and/or behavioral comorbidities, including attention deficit hyperactivity disorder (ADHD), obsessive-compulsive behavior (OCB) or disorder (OCD), anxiety, depression, and problems with impulse control and sleep (1, 3–8).

TD was first described by a French physician Dr. Jean-Marc Gaspard Itard in 1825 (9) to report patients with involuntary motor tics with echolalia and coprolalia, and then named as Gilles de la Tourette syndrome (often shortened as Tourette syndrome, TS) by Dr. Jean-Martin Charcot in 1885 after his resident Dr. Georges Gilles de la Tourette (10). However, subsequent clinical experience has found that there are more patients with involuntary tics without echolalia and coprolalia, thus only TD. Notably, TS only represents the most severe form of TD in the current DSM-5 diagnostic criteria (2); but it is still often used to describe TD in the Western clinical practice and literature (3). Interestingly, some experts in the field recently suggested that a unified diagnosis of tic spectrum disorder (TSD), similar to autism spectrum disorder (ASD) in DSM-5, would be more appropriate and practical to diagnose individuals with a primary TD; while also reducing stigmatization by discarding the ‘Tourette Syndrome’ title” (11).

TS diagnosis was introduced to China in the early 1980s after several Chinese pediatric neurologists and psychiatrists first went abroad as visiting scholars and learned about the Western diagnosis and treatment of TD (12). In the Chinese literature and clinical practice, “tic-coprolalia syndrome” was initially used as an equivalent for TS, but “tic disorder” has been gradually used to replace “tic-coprolalia syndrome”, to include the majority of tics without coprolalia, as well as avoiding stigmatization of children suffering from “tic-coprolalia syndrome”. In Chinese culture, the manifestation of tics is considered as embarrassing, and coprolalia is socially unacceptable. However, this situation has ameliorated in recent years through the campaign of public education, such as public educational programs and social media spread of medical knowledge and scientific facts about TD (12). For example, August 3 has been set as the TD Day in China since 2012 to raise the public awareness of TD.

Epidemiological studies have shown that TD is equally common across all races, ethnicities, and populations, with transient and mild tics affecting as many as 20% of school-age children (13, 14); while chronic TD and TS affect 0.3–5.0% and 0.3–1.0% of school-age children, respectively in various Western populations (3, 6, 8, 13–16). TD is likewise a quite common neuropsychiatric disorder in the Chinese pediatric population as well (12). A meta-analysis of 13 epidemiological studies between 1992–2010 in China showed that the prevalence of combined TD, provisional TD, chronic TD, and TS was 6.1% (95% CI: 0.036–0.100), 1.7% (95% CI: 0.009–0.031), 1.2% (95% CI:0.007–0.022), and 0.3% (95% CI: 0.001–0.008), respectively (17), which are comparable with the prevalence reported in other world populations. Currently, more than 20% of the 1.4 billion people living in China are in the 0–18 age bracket (https://www.unfpa.org/data/world-population-dashboard). Therefore, it is estimated that more than 10 millions of children and adolescents in China suffer from some mild form of TD, and up to 1 million with TS (12, 17).

In China, children with TD are often mainly under the care of general pediatricians and pediatric neurologists. Pediatric psychiatrists are usually consulted for or in charge of more severe cases with mental comorbidities (12). However, presently child psychiatry is still a discipline in its nascent stage in China with less than 500 available qualified pediatric psychiatrists, and most of them practice in big cities, such as Beijing, Shanghai, Guangzhou, and Wuhan (12). Nevertheless, there is no strict tiered referral healthcare system in China and the patients with TD could seek medical service anywhere in China.

As one of the main bodies of physicians treating children with TD in China, the Chinese Child Neurology Society (CCNS), with ~2,300 members from 31 provinces and regions (18), formed a consortium working on TD and reached an agreement in 2013 to jointly improve the diagnosis and management of TD/TS nation-wide; and subsequently developed a Chinese version of expert consensus on diagnosis and treatment of TD in children (19), and further revised it in 2017 (20). Recently, the Chinese Child Neurology Society Tic Disorders Consortium have collectively reviewed the current updates in the field and revised the latest Chinese version of TD diagnosis and treatment, based on the most recent recommendations from the American Academy of Neurology for treatment of TD/TS, and feedback from colleagues in China. Here we present an updated English version of this Chinese experts’ opinions on current status of TD, and its diagnosis and treatment in China, which are mainly established on the commonly adopted Chinese national (19–22) and international practices and standards (15, 16, 23–28).

## Pathophysiology of TD

TD used to be considered as a mysterious illness, and the cause was mostly unknown (9, 10). However, decades of clinical observation and basic research have suggested that TD is a spectrum of neurodevelopmental disorders. The pathogenesis of TD could be due to a combination of genetic, immunological, psychological, and environmental factors. The links between the pathophysiology and clinical symptoms probably lie in the disinhibition of the cortical-striatum-thalamus-cortical circuits (4, 29–31). An imbalance of inhibitory–excitatory signals in these circuits is considered as the molecular mechanism to produce the tics and related symptoms. For example, overactivity of striatal dopamine or postsynaptic dopamine receptor hypersensitivity could produce tic symptoms (8, 29, 31, 32). Multiple neurochemical and neurotransmitter abnormalities have been implicated in TD/TS, most notably dopaminergic, adrenergic, GABAergic, and glutamatergic pathways (3, 4, 31). More recently, histaminergic (33, 34) and endogenous cannabinoid pathways have been associated with TD/TS through genetic, pharmacological, and brain imaging studies as well (35, 36).

Furthermore, recent extensive genetics, neuroimaging, and neurophysiology research have demonstrated that TD/TS with or without comorbidities of ADHD and or OCD are not distinct disorders but instead arise from common neurodevelopmental abnormalities of parallel cortical-striatal-thalamo-cortical circuits, which regulate initiation, selection, execution, learning, and reinforcement of intended movements, thoughts, behaviors, and moods (30, 31, 37). While tics could arise from dysregulation of the sensorimotor and oculomotor loops, OCB/OCD symptoms may stem from dysregulation of the anterior cingulate and lateral orbitofrontal loop, and ADHD symptoms could be due to dysregulation of the dorsolateral prefrontal loop (30, 31, 37).

Previous studies have suggested that TD/TS is highly inheritable and the heritability is up to 0.77, but no definitive TS causal gene has been identified (38, 39). Recently, a largest-ever genome-wide association and family co-segregation studies with 4,819 TS case subjects and 9,488 control subjects have found only one significant locus (***FLT3*** on chromosome 13, rs2504235, with odds ratio = 1.16), and TS polygenic risk scores could significantly predict both TS and tic spectrum disorders status in the population-based sample (40). Moreover, a meta-analysis of eight psychiatric disorders in 232,964 cases and 494,162 controls, including independent ADHD, OCD, and TS samples, detected 109 loci associated with at least two psychiatric disorders, and 23 loci with pleiotropic effects on four or more disorders. These loci are enriched among genes highly expressed in the brain and play prominent roles in neurodevelopmental processes (41). These findings indicate that TD/TS may be highly polygenic in nature, and TD/TS and its comorbidities may share some overlapped genetic origins, pathogenic pathways, and underlying neural circuits.

## Clinical Characteristics of TD

### Age of Onset and Sex Differences

Tics mostly begin before 18 years of age, typically between 4–8 years old, and the mean age at onset is around 6 years old (42). The tics increase in severity to a peak around 10–12 years old, and then gradually decrease and some remit in late adolescence and young adulthood (4, 6–8, 15, 16, 43).There are rare cases of adulthood onset TD, which is not within the scope of this proposal.

TD and its various subtypes are more common in boys than girls, and the ratio of male to female is estimated to be 3–4:1 (4, 6–8, 14, 42). In a meta-analysis of TD in China, the boy to girl ratio ranged from 2.22 to 3.68 for transient TD, 1.57–2.79 for chronic TD, and 2.17–10.6 for TS, which are in line with the global reports of sex difference in TD/TS (17).

### Clinical Manifestation of Tics

The word “tic” is evolved from the French word “tique”, meaning a sudden, aimless, fast, and rigid muscle contraction (9, 10). Tics are divided into motor tics and vocal tics. Motor tics are rapid contraction of the fingers, face, neck, shoulders, trunk, and limbs. Vocal tics are the contraction of the oropharynx, throat, and respiratory muscles, and the sound is produced through the airflow in the nose, mouth and throat.

Motor tics or vocal tics can be further divided into two categories: simple and complex, depending on the duration of tics and part(s) of body and group(s) of muscles involved. Simple tics involve brief activation of single muscles or a localized muscle group and manifesting as simple movement or sound; while complex tics activating more muscle groups and manifesting as a goal-directed or purposeful-like movements, or sounds of word or phrase.

Practically any body-muscle may be involved in tics, and there is wide variability in tic phenomenology. Nevertheless, certain tics occur much more frequently than others, and the most common tic is eye blinking. Some descriptive examples of tics are given in **Table 1**, and some video demonstrative examples could also be found in the supplementary online files from the reference (31). Tic symptoms usually start from the face, gradually spread to the muscles of the head, neck, and shoulder, and then to the trunk and upper and lower extremities.

**Table 1:** Manifestation and classification of tics.

Tic Type	Simple Tic	Complex Tic
Motor Tic	Blink of eye/oblique eye, frown, eyebrows, open mouth, loll tongue, tapir mouth/crooked mouth, lick lips, crumpled nose, nod/raise/shake/swivel head, torticollis, shrug shoulders, move fingers/toes, rub hands, clench fist, move wrists, lift/stretch/internal rotate arms, stretch/shake legs, step/pedal foot, extend/bend knees, extend/bend coxa, lift chest, hold abdomen, twist waists, and so on.	Lift eyebrows and wink, make faces, eyeball rotate, knob fingers, swing/clap hands, wave arms, stab action, flick limbs, hit chest with fists, bend waist, mandible touch the knee, twist trunk, move up and down, squat, kneel posture, kick legs, knee joint, stamp foot, jump, hop, throw, beat, touch, sniff, touch the hair, walk in circles, walk backwards, and so on.
Vocal Tic	Single-tone, sniffing, clearing throat, roaring, humming, coughing, squeaky sound, screaming, shouting, grunting, spitting, whistling, sucking, barking, tweeting, and so on.	Single word/phrase/clause/sentence, repeat single word or phrase, repeat sentence, imitate speech, obscene language, and so on.

Tics are mostly involuntary but could also be voluntarily held temporarily, particularly in older children. However, voluntary tic suppression could result in a tic “buildup”, followed by a sense of relief when the tics are finally carried out (44). Sometimes in older children with longer disease course, after motor tic or vocalization, another action could quickly occur in an attempt to hide or disguise the tics, making the clinical manifestations more complex and challenging to recognize (20).

Forty to fifty-five percent of children report a premonitory urge before motor tic or vocalization, which is an urge-for-action to a perceived local sensory stimulus or sensation or discomfort. Such a premonitory urge could manifest as local pressure, itching, pain, hot, or cold sensation, or other strange feelings, which are also called sensory tics (32, 45, 46). Sensory tic is considered as a premonitory symptom that will disappear after the tics, especially in older children (4, 32, 46). Motor tic or vocal tic could be related to relieving such premonitory urge or local discomfort. Premonitory urge is a characteristic feature of TD, and its awareness and control increase with age (47).

Echopraxia (involuntary repetition or imitation of another person’s actions), echolalia (repetition of other people’s vocalizations), and palilalia (repetition of the last word or phrase said by the patient) are present in some patients with TD. However, it is noteworthy that echophenomena (echopraxia and echolalia) are essential developmental elements in social learning up to the age of 28–36 months. So only when their persistence beyond this developmental age should prompt diagnostic consideration for a neuropsychiatric disorder, including TD (48). Copropraxia, the involuntary making of obscene gestures, and coprolalia, inappropriate, and out-of-context swearing, are complex forms of motor and vocal tics, respectively. They have been strongly associated with TD/TS, but relatively uncommon in clinical practice, affecting less than 30% of TD/TS patients (47, 49).

### Clinical Course of TD

The tics in one individual can change from one form to another, and new forms of tics could emerge during the disease course, but usually manifest as some specific stereotype during a particular time period (50). The frequency and intensity of tics could also fluctuate significantly during the disease course, and new tic symptoms can replace old tic symptoms or superimpose on old tic symptoms.

Tics usually occur in bouts, and tic symptoms often wax and wane during the disease course (4, 43), and they can also be aggravated or mitigated by some stimuli. For example, common factors exacerbating tics include stress, anxiety, anger, shock, excitement, fatigue, infection, and being reminded, while common factors reducing tics include attention concentration, relaxation, emotional stability, and sleep (43, 45). Exercises, especially those involving fine motor movements, such as dancing or sports activities, are often associated with tic attenuation as well (43, 45).

### Comorbidity of TD

About half of the children with TD and more than 80% of patients with TS suffer from at least one comorbid psychopathological or behavioral disorder(s) and about 60% TS patients suffer from two or more, which are known as comorbidities (4, 42, 43, 51), e.g., ADHD, OCB or OCD, learning difficulties, anxiety, depression, sleep disorders (52, 53), self-harm behavior or self-injurious behavior (SHB or SIB) (54), conduct disorder (55), rage attacks, or explosive outbursts (32, 56–58). Among them, ADHD is the most common comorbidity, followed by OCD, affecting approximately 50–60% and 36–50% of the patients with TD/TS, respectively (4, 43). There is also a sex difference in the incidence of TD/TS comorbidity. Usually, ADHD, learning difficulties, conduct disorder, and rage attacks are more common in boys, while OCD and SHB/SIB happen more often in girls (4, 43, 47, 51). Comorbidity increases the complexity and severity of TD (4, 43, 47), affects the healthy development of children’s learning, social adaptation, personality, and psychological quality, and adds much more difficulties and challenges to the diagnosis, treatment, and prognosis of the illness (59).

## Diagnosis of TD and Clinical Assessments

Extensive clinical observation and research have shown highly variable clinical manifestations, severity, and comorbidities in patients with TD. Such high clinical variability has created significant challenges in clinical diagnosis and management, as well as for clinical research. In the last two decades, better understanding of this group of disorders and collaborative efforts have facilitated the development of standardized diagnostic procedures and criteria for tics and related disorders. For example, the DSM-IV-TR in 2000 (60), the Chinese Classification of Mental Disorders 3^rd^ Edition (CCMD-3) in 2001 (61), the DSM-5 in 2013 (2), and the ICD-11 in 2018 (62) all have specific criteria for the diagnosis of TD and related medical conditions. The CCMD-3, DSM-5, and ICD-11 diagnostic criteria for tics are almost the same. Currently, the DSM-5 is mostly used in clinical practice around the world, including China. Notably, the older versions of diagnostic criteria had a discrepancy in terms of tic-free period regarding the 1-year duration for diagnosis of chronic TD and TS, i.e., CCMD diagnostic criteria specified no remission period of >2 months in 1-year period for chronic TD and TS. In comparison, the DSM-IV-TR diagnostic criteria specified no remission period of > 3 months, which could be a contributing factor for variation of prevalence in the previous reports. Nevertheless, the current commonly used DSM-5 criteria have no limitation on remission or symptom-free periods.

**Figure 1:**
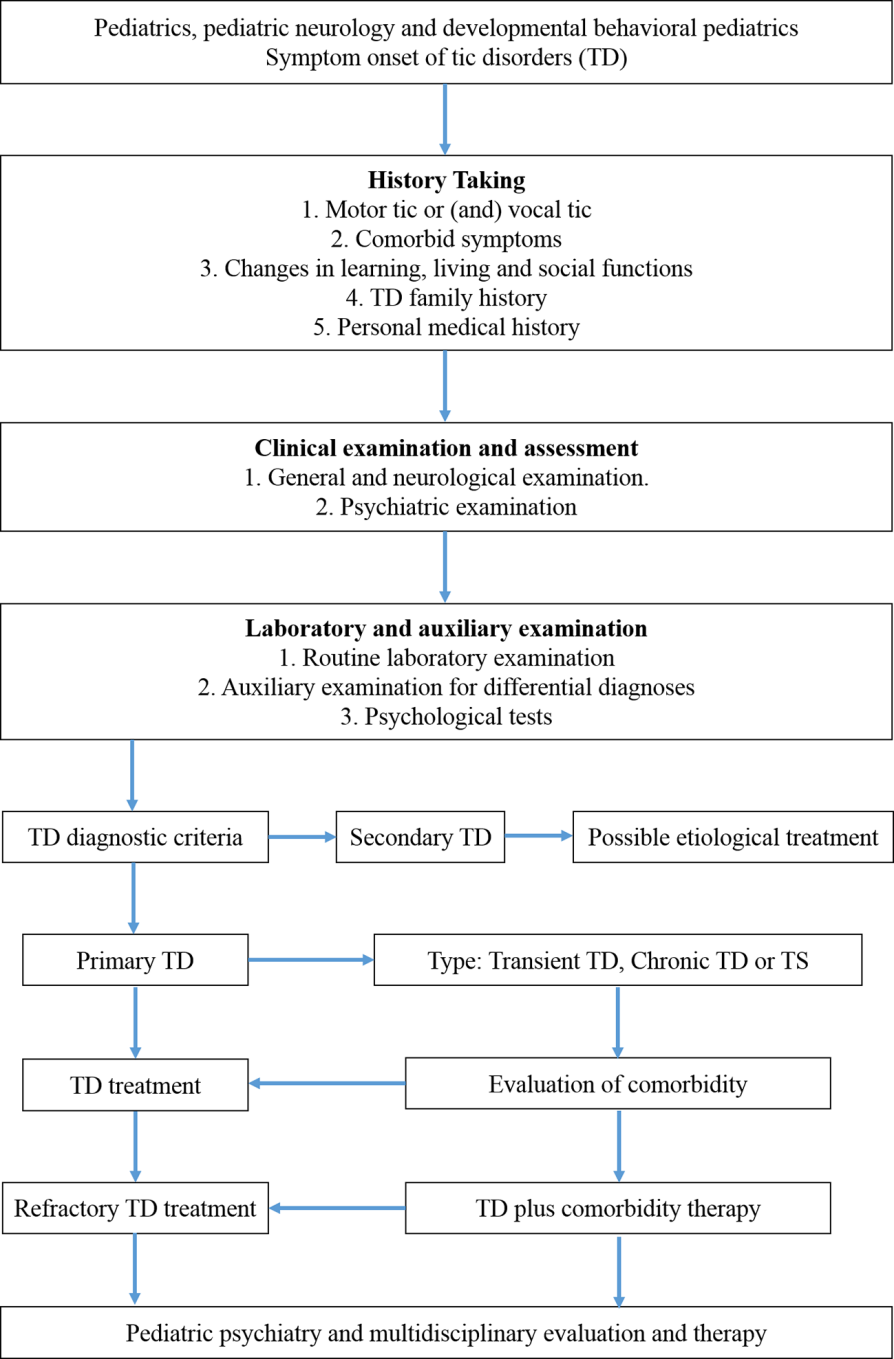
Diagnostic and Treatment Roadmap of TD.

At present, descriptive clinical diagnostic methods are mainly used to identify children with tic symptoms and associated mental and behavioral manifestations. Therefore, a detailed inquiry of the medical history and careful observation of the tic manifestation and its associated abnormal cognition and behaviors are the prerequisite for a correct diagnosis. A thorough medical history should include mother’s medication during pregnancy, birth history, early development, and past medication use by the patient, etc. plus a complete psychosocial and family history to detect psychiatric and or neurological conditions in relatives. A thorough physical, neurological, and psychiatric examination are critical to identifying any potential causal factor, and symptoms and signs of accompanying medical conditions. Please refer to **Figure 1** for essential diagnostic steps.

### International Diagnostic Criteria

According to the clinical characteristics and course of the illness, TD can be divided into three types according to DSM-5, i.e., Tourette syndrome (TS), chronic TD, and provisional TD.

**TS:** (1) both multiple motor and one or more vocal tics, but the motor and vocal tics do not necessarily appear at the same time. (2) onset before age 18. (3) after the first onset of tics, the frequency of tic can increase or decrease, while the duration of the tic symptoms is more than one year. (4) tic symptoms are not caused by certain drugs or substances or other medical conditions.
**Chronic TD, previously known as persistent TD:** (1) single or multiple motor or vocal tics, but not both motor and vocal tics at the same time. (2) onset before age 18. (3) since the first onset of tics, the frequency of tics can increase or decrease, while the duration of the disease is more than one year. (4) tic symptoms are not caused by any medication or substance or any other medical conditions. (5) fail to meet the diagnostic criteria for TS.
**Provisional TD, previously known as transient TD:** (1) single or multiple motor and/or vocal tics but not both motor and vocal tics at the same time. (2) onset before age 18. (3) the duration of tics is less than one year. (4) tic symptoms are not caused by any medication or substance or any other medical conditions. (5) fail to meet the diagnostic criteria for chronic TD or TS.

There is a certain continuity between the three types, with transient TD can develop into chronic TD, while chronic TD can also transit into TS. Some patients do not fall into any of the above categories; they belong to other TD, such as adult-onset TD or late-onset TD, and any other unspecified TD.

Refractory TD is a new concept gradually formed in pediatric neurology/psychiatry in recent years, and there is no clear definition yet. It is generally accepted to consider as refractory TD when a severe case of TS has been treated with classical anti-TD medications, such as tiapride and haloperidol or aripiprazole, for more than 1 year without satisfactory results (63).

### Differential Diagnoses and Auxiliary Exams

First of all, tic symptoms should be differentiated from epileptic seizures, substance or medication-induced dyskinesia, chorea, dystonia, to name a few (32, 64). The presence of a premonitory urge with relief following the tic movement, the ability to suppress the tic movement, as well as the waxing and waning pattern support the diagnosis of TD. Furthermore, tics occur when the motor function of the involved muscle is normal; and tics are sudden, brief, and repetitive, and happen temporarily and episodically (32, 64).

Secondly, the majority of TD cases are primary TD or idiopathic, in which tics are the main clinical manifestation and no direct cause could be identified. Hence there are no specific biomarkers nor diagnostic tests for primary TD. Neurologic examination is usually normal for patients with primary TD. However, “soft” neurologic signs may present, including impaired fine movement coordination and motor restlessness, especially in children with ADHD (31).

In general, electroencephalogram (EEG), neuroimaging, psychological test and laboratory examination are not required to support the diagnosis for primary TD. The results of such an examination could show nonspecific abnormalities; and they are mainly used to assist the diagnosis of comorbidity or exclude the possibility of other diseases. In a small number of children with tic-like symptoms, EEG could show background slow waves or asymmetry, or paroxysmal epileptiform discharges, which is helpful to identify any active brain pathology or concomitant or mistaken seizure disorders. Video-EEG is commonly available in most major cities of China with affordable cost therefore it could be considered a routine test to exclude epileptic seizures and other neurological conditions (20).

Skull CT or MRI examination in some patients with TD could show smaller caudate nucleus, slightly thinner frontal and occipital cortex, mild ventricular enlargement, or deeper lateral fissure, and other nonspecific structural changes (65). Therefore, brain imaging examination could mainly be used to exclude any structural lesion of the basal ganglia and other relevant brain regions if suspected (65).

However, it is important to keep in mind that a variety of medical conditions and acquired factors could also cause tics or tic-like symptoms (8, 29, 46, 64). According to the previous studies, the following medical conditions and disorders could present tics or tic-like movements as the main or part of the clinical manifestations (8, 29, 46, 64): (1) genetic syndromes, in which tics or tic-like symptoms are only part of, but not the primary clinical manifestation, such as Down’s syndrome, Fragile X syndrome, tuberous sclerosis complex, and neuroacanthocytosis; (2) infectious diseases, such as streptococcal infection, encephalitis, neurosyphilis, Creutzfeldt-Jakob disease; (3) intoxicating factors, such as carbon monoxide, mercury, or bee poisoning; (4) medication side effects, such as methylphenidate, pemoline, amphetamine, cocaine, carbamazepine, phenobarbital, phenytoin, and lamotrigine; (5) other factors, such as stroke, and head trauma.

In such above-mentioned situations, the patients will present repetitive, patterned, but aimless and inattentive tics or tic-like movements, or seemingly stereotypy movements. Careful observation, medical history and physical examination could differentiate them from primary TD/TS (32, 66). A secondary TD should be suspected when tics present in much older children, start abruptly, or rapidly worsen over days to weeks, or occur in patients with other neurologic signs or symptoms. Systematic screening and specific auxiliary tests should be ordered to exclude the above-mentioned causes if suspected. The laboratory blood tests of antistreptolysin “O” (ASO), erythrocyte sedimentation rate, rheumatoid factor, virus antibody, trace elements, and ceruloplasmin are helpful to identify some common causative factors or for differential diagnoses (46, 67). Consultation or referral to specialised health professionals could help solving some complicated cases of secondary TD with some rare primary disease conditions.

### Tics Severity Assessment

The severity of tics and its associated comorbidities and functional impairment are also highly variable. Tics could be simply divided into mild, moderate, and severe cases based on simple clinical observation. Mild cases refer to light tic symptoms, which do not affect children’s normal lives, learning, or social activities. Moderate cases refer to frequent tic symptoms, which somehow interfere with children’s normal functions and social activities. Severe cases refer to very frequent tic symptoms, which significantly impair children’s lives, education, and social activities.

Nevertheless, it is highly recommended to use a standardized instrument to objectively, quantitatively, and systematically evaluate the severity in order to monitor the disease course and treatment effect. There are several different tools that have been developed to measure the severity of TD and its associated psycho-social-behavioral comorbidities and impairments (32), such as the Gilles de la Tourette Syndrome Health-Related Quality of Life Scale (68) and the Premonitory Urge for Tics Scale (69). One of the most commonly used tic severity measurements is the Yale Global Tic Severity Scale (YGTSS) (70).

YGTSS is based on a semi-structured clinical interview and consists of three parts. The first part consists of checking items of motor/vocal tic symptoms. The second part is a score-system to assess the severity of motor and vocal tics separately in five dimensions, including tic numbers, frequency, intensity, complexity, and interference. The third part is the scale of functional impairments in self-esteem, social interaction, study, or work of children with TD. The total score of YGTSS (maximum rating 100) is obtained by summing up the scores of motor and vocal tics and functional impairment. TD cases with less than 25 of YGTSS total scores are considered as mild, 25–50 scores as moderate, and more than 50 scores as severe.

Besides, the number of comorbidities is also highly associated with the overall severity of TD, with more comorbidities, more severe the cases are (46). Repeated measures of the YGTSS can help systematically monitoring the disease course and evaluating response to treatment. A recent study has further proved its utility in assessing tic severity in children and adults with some minor revisions, i.e., YGTSS-Revised (links.lww.com/WNL/A423) (71).

### Diagnosis of Comorbidity

In any case, a careful evaluation of the common comorbidities is an essential component of the TD assessment. MINI Kid 5.0 (Mini-International Neuropsychiatric Interview for Children and Adolescents) is a short, structured diagnostic interview for DSM-IV and ICD-10 psychiatric disorders in children and adolescents (72). It has been translated and validated in Chinese language and is highly recommended by the Chinese pediatric psychiatric experts for diagnoses of psychiatric comorbidities of TD (73).

#### TD/TS With ADHD

ADHD is characterized by an enduring pattern of developmentally inappropriate inattention or hyperactivity and impulsive behavior. ADHD is reported to affect about 50% (range of 21%–90%) of referred patients with TD/TS, compared to 2%–12% in the general pediatric population (5, 74). ADHD symptoms (inattentiveness, hyperactivity-impulsivity, or both) usually precede the onset of tics by 2–3 years (5, 74). Comorbid ADHD may contribute to behavioral disturbances, such as aggressiveness, disruptive behaviors, poor school performance and social adaptation, and problems with executive function, as well as increased emotional problems and functional impairments (5, 74). Therefore, a coexisting ADHD and its health burden should be screened for every patient with TD (32) through MINI Kid 5.0 (72, 73). Thorough evaluation could be performed by using pediatric ADHD rating scales within the Child Behavior Checklist (CBCL), and the Children’s version of the Connors ADHD Rating Scale (CAARS) if needed (5, 32, 45).

#### TD/TS With OCD

OCD is characterized by the occurrence of obsessions, which manifest as recurrent and intrusive thoughts, ideas, images, or impulses; and the occurrence of compulsions, which are repetitive behaviours or mental acts sought to prevent or reduce anxiety or distress. The DSM-5 criteria for OCD require that obsessions, compulsions, or both occupy at least 1 h per day or cause significant clinical distress or functional impairment.

A lifetime comorbid diagnosis of OCD is present in about 50% of patients with TS (5). To a lesser degree, OCB usually presents as a need for order or routine, and a requirement for things to be symmetric or in specific order or pattern, e.g., repeated checking or reordering or counting, rituals, and forced touching (5). OCB occurs in 20%–60% of patients with TS, compared to 0.5%–3.6% of healthy children and adolescents. OCB/OCD often emerges during early adolescence, several years after the onset of tics, frequently accompanied by a higher frequency of aggressive behaviors (5). The most recommended instrument to capture the full range of obsessive and compulsive symptoms and assess its severity in children is the Children’s Yale-Brown Obsessive–Compulsive Scale (CY-BOCS in children), entailing 58–80 items on obsessive and compulsive symptoms and 10 items on obsessive and compulsive severity (32).

#### TD/TS With Anxiety and Depression

The presence of generalized anxiety disorder in subjects with TD/TS has been reported in the range from 19%–80%, with a high-risk period for anxiety issues starts at age 4, and a high-risk period for mood disorders begins at age 7 (47, 75). The presence of depression in patients with TS has been positively correlated with an earlier onset, greater severity, and a longer duration of tics (31, 45, 64, 75). Anxiety and depression should be routinely screened in children and youth with TD/TS by MINI Kid 5.0 (72, 73), and properly assessed using the Multidimensional Anxiety Scale for Children (MASC) and the Children’s Depression Inventory (CDI) when the symptoms are prominent and intervention is needed (75).

#### TD/TS With Disruptive Behaviors and Potentially Life-Threatening Symptoms

Disruptive behaviors, including episodic outbursts, rage, aggression, and impulse control problem, are common in patients with TD/TS, e.g., episodic behavioral outbursts and anger control problems have been reported in 25%–70% of TS populations (31, 45), which should be recognized during history taking and considered when planning for intervention and therapy.

The risk of premature death was reported to be higher among individuals with TD (mortality rate ratio, 2.02; 95% CI, 1.49–2.66) and with TS (mortality rate ratio, 1.63; 95% CI, 1.11–2.28), compared with controls in a large population-based prospective cohort study in Denmark (76). When having complex and greater severity of motor symptoms, plus the presence of two or more behavioral comorbidities, particularly OCB/OCD, the patients could result in tic-related injuries, self-injurious behaviors (SIB), uncontrollable violence and temper, and suicidal ideation/attempts (31). Notably, copropraxia and coprolalia have been reported highly associated with SIB in patients with TD/TS (47). Such high-risk symptoms and behaviors need to be recognized during psychiatric assessment, and these patients need immediate medical attention and intervention to prevent severe consequences (54, 77).

## Therapeutic Approaches

There have been some consensus TD/TS treatment recommendations in the past decade in the Western countries, based on some experts experience and the clinical evidence, such as the European Clinical Guidelines for Tourette syndrome and other TD (15, 23, 24, 32, 78), the Canadian guidelines for the evidence-based treatment of TD (16, 25, 26), and the most recent American Academy of Neurology Practice Guideline Recommendations for Treatment of Tourette syndrome and Chronic TD (28).

The establishment of an effective therapeutic plan requires careful initial assessment of tics, determining the presence of co-occurring psycho-social-behavioral issues, and clarifying the resulting impairment of each issue. Many children and adolescents with TD do not require intervention or treatment for their tics if their tics do not interfere with daily life or school activities. In China, there are also variations in terms of availability of cognitive-behavioral therapy (CBT) and pharmacological treatments, as well as clinicians’ experiences to justify the clinical guidelines in different places and conditions. Therefore, treatment decisions should be based on an individual patient’s needs, available resources, experience of the treating clinician, guided by the recommendations from the experts and professional organizations in the fields.

Target symptoms should be identified before treatment starts, i.e., the most influential symptoms on patients’ daily life, study, or social activities. Tics are usually the main target symptoms of treatment, while the target symptoms of some children could be more prominent comorbid symptoms, such as hyperactivity, impulsiveness, obsessive compulsion, and so on.

For children with mild TD, medical education and psychological support could be offered first or only, and a watchful waiting period with regular follow-ups should be appropriate. The principle of treatment for moderate to severe TD is similarly to try non-pharmacological intervention first, and behavioral therapy could be combined with pharmacological treatment. Nevertheless, medical education and psychological support should be provided throughout the entire treatment course. Please refer to **Figure 1** for therapeutic order and steps.

### Education and Support of Patients With TD and Their Families

Before to and at the same time of the active treatment of TD/TS, we would first recommend medical education and psychological support to the patients, as well as their parents, peers, and teachers in school and communities (24, 28, 79, 80), which could be in the forms of parent management training, parent-child interaction therapy, parent-school teacher interaction etc. It is essential to inform and educate patients and their parents that for most people with TD, the tics subside on their own by the end of adolescence. This fact about TD could lead to a much more conservative therapeutic need and approach.

#### Parent Management Training

Parents could be taught to record short videos of the children’s symptoms at home and show them to the doctors at the clinics so that the doctors could have a better assessment of the condition. Parents could be encouraged to face the diagnosis of TD/TS with their children instead of being embarrassed and trying to deny or hide or finding “excuses” for the tics symptoms for their children. Parents could also be advised to reassure the children with TD/TS to interact confidently with their classmates and people around them so their social adaptability could be improved. They could also motivate the children to actively take part in physical and social activities, instead of over-protecting the children by keeping them inside and isolated from others, which is common in the Chinese culture, particularly in the current generation of one-child families in China (12). The parents could also be instructed to carefully observe with the children the conditions and factors that could provoke or increase the tic symptoms, and subsequently avoid such “risk factors”.

The Chinese TD/TS Association, which is a non-governmental, non-profit organization, has built a platform for health education, patient-physician and patient-patient interaction, and information exchange among physicians, patients with TD, and their families. It is a very useful resource for information and support for patients with TD/TS and their families.

#### School and Educational Support

Parents could also communicate more often with school-teachers to help them better understand the medical condition so that the children with TD/TS could avoid being punished for “unexpected or uncontrolled movements” and could have reduced academic work-load to lower their stress level. School teachers could also help educating other students not to laugh at, isolate, and stigmatize the children with TD/TS.

We would also recommend special educational support for children with TD/TS, and particularly those with problems of learning, social adaptation, and self-esteem. We believe such special support could promote rehabilitation and help children return to healthy life. Most children with mild TD and good social adaptability can achieve effective results through psychological education and support only.

### Cognitive Behavioral Therapy of TD

Cognitive behavioral therapy (CBT) and/or pharmacological interventions should be considered in addition to psychoeducation for patients with TD who have clear impairment associated with the tics. There is no clear consensus on what constitutes an indication to start treatment in TS. However, the European guidelines published in 2011 (15) recommends starting behavioral or pharmacological treatment for tics in the following situations: (1) subjective discomfort otherwise requires other treatment; (2) social impairment; (3) emotional difficulties; (4) functional disabilities. These situations mainly correspond to moderate to severe TD.

Behavioral therapy is an effective means to reduce tic symptoms and comorbidity, and to improve social function (79, 81, 82). Multiple behavioral interventions have been developed for the treatment of TD/TS and its associated comorbidities, including habitual reversal training (HRT), effective prevention of exposure, relaxation training, positive reinforcement, self-monitoring, regression exercise, to name a few (3, 79). The most commonly used one is comprehensive behavioral intervention for tics (CBIT) (3, 29), which trains patients to become aware of their tics and teaches them specific behavioral strategies to reduce tics. CBIT has been shown superior to supportive psychotherapy for children aged 10–17 years with TS and considered as first-line therapy when available, including those with comorbid OCD and ADHD (3, 27–29).

However, behavioral therapies are unlikely to be helpful in very young children (aged 9 years and younger), who have limited cognitive function to recognize and control pre-impulses that are the core of behavioral therapy; or in children with severe, untreated ADHD, who may have difficulties sustaining engagement in therapy. Furthermore, behavioral interventions are resource-intensive and require the presence of highly skilled clinicians, typically psychologists, occupational therapists, or specially trained physicians, and their significant time commitment (83). Due to the shortage of such trained professionals, as well as lack of experience and confidence among clinicians and parents, widespread implementation of behavioral interventions of TD still faces challenges in China (84). Currently, it is only available at certain pediatric mental health centers in some big cities’ of China, e.g., Beijing, Shanghai, and Wuhan. Nevertheless, behavioral therapies are much safer than pharmacological medications and have been proven effective in older children with TD/TS (24, 26–28). Therefore, it has been highly recommended by the Western experts and guidelines, and it could be gradually introduced and implemented in some centers in China with a large volume of TD/TS patients and available resources.

### Pharmacological Treatment of TD

For children with moderate to severe TD that affects daily life, school, and social activities, and when psychological education and behavior therapy are not effective or unavailable, pharmacological therapy is needed (28, 85). The patients and their parents need to understand at the beginning of pharmacological therapy that the outcome of the medication(s) is unlikely to be completely tic-free but rather to improve the control and reduction of tic severity. The currently available medications could reduce tics by over 60%, such as aripiprazole in 60.2% (86–88), tiapride in 76.0% (27, 59). In general, a two-tiered medication choice and multi-stage treatment course are recommended, with the use of first-line medications for milder tics and the use of second-line medications reserved for more difficult cases. Therapeutic agents should start with monotherapy at its lowest effective dosage and gradually increased as needed. It is inappropriate to change the medication(s) or to discontinue the medication(s) too early or abruptly.

#### Course of Pharmacological Treatment

It is highly recommended that the pharmacological treatment of TD will take a gradual process and divide into multiple stages with careful evaluation at each step (19, 20). The complete course of treatment usually takes 1–2 years. If symptoms reoccur or aggravate at any time during the course, then return to the previous step or resume the process from the beginning.

1. Acute treatment period: Actively control the symptoms and shorten the course of the illness. Starting from the minimum dose, slowly increase (1–2 weekly added) to target treatment dose. The course of treatment is dependent on the patient’s response to the medication until satisfactory result is achieved.
2. Consolidating treatment period: Consolidate therapeutic effect, prevent relapse, and promote social function recovery. After the tic symptom is mostly under control, the same dosage should be continued for at least 1–3 months.
3. Maintenance treatment period: Prevent relapse, maintain good daily function, and improve the quality of life. After the consolidation period, if the condition is well-controlled, the treatment should remain for 6–12 months, and the maintenance dose is generally 1/2–2/3 of the maximum dose previously used.
4. Medication withdrawal period: After the maintenance treatment, if the symptom(s) are well under control, the medication can be gradually withdrawn; and the withdrawing period should be gradual and last at least 1–3 months.

#### Medication Options

Some recommended medications for the treatment of pediatric patients with TD in China are shown in **Table 2**, which are based on our clinical experience and availability of the drugs in China, including two proprietary polyherbal Chinese medicines that have been approved by the Chinese National Administration of Traditional Chinese Medicine (TCM), and are recommended as the first-line TCMs for pediatric patients with TD by the National Guideline of TCM for Pediatric Diagnosis and Treatment through a TD Expert Committee (22) (please also refer to the *Traditional Chinese Medicine Treatment of TD in China*). The choice of medication is often driven in part by the patient’s comorbidity profile, and treatment sometimes needs to target multiple symptoms, for example, tics plus hyperactivity, or anxiety, or compulsion. Every patient need be carefully followed up and has periodic evaluation and check-up to assess medication efficacy, side effects, and the need for continued therapy.

**Table 2:** Recommended medications in the treatment of TD.

Recommendation References	Drug Name	Type	Mechanism of Action	Initial Dose	Therapeutic Dose ^a^	Common Side Effects
First-line Med (20, 89, 90)	Tiapride	Antipsychotic, typical neuroleptic	D2 receptor blockade	50–100 mg/d	100–600 mg/d	Somnolence, gastrointestinal reactions
First-line Med (20, 23, 86, 88–91)	Aripiprazole	Antipsychotic, atypical neuroleptic	Partial agonist of dopaminergic (D2, D3, and D4 receptor) and serotonergic (5-HT1A and 5-HT2C) receptors	1.25–5.00 mg/d	2.50–20.00 mg/d	Somnolence, weight gain, gastrointestinal reactions
First-line Med (TD+ADHD) (20, 23, 92–94)	Clonidine ^b^	Alpha agonist	α2 adrenergic receptor agonist	1.0 mg/w	1.0–2.0 mg/w	Somnolence, dry mouth, dizziness, headache, fatigue, occasional orthostatic hypotension, and bradycardia
First-line Med (22, 95)	Changma Xifeng Tables	TCM ^c^	Unknown	0.53–1.59 g/d	1.59–4.77 g/d	No obvious adverse reaction
First-line Med (22)	Jiuwei Xifeng Granule	TCM	Unknown	6.0–12.0 g/d	12.0–24.0 g/d	No obvious adverse reaction
Second-line drug (20, 23, 89, 90)	Haloperidol	Antipsychotic, typical neuroleptic	D2 receptor blockade	0.25–1.00 mg/d	1.00–6.00 mg/d	Somnolence, extrapyramidal symptoms, increased appetite, and hepatic insufficiency
Second-line drug, off-label use (20, 23, 89, 90)	Risperidone	Antipsychotic, atypical neuroleptic	5-HT2 receptor antagonist at low doses andD2 antagonist at high doses	0.25–1.00 mg/d	1.00–4.00 mg/d	Weight gain and extrapyramidal response
Second-line drug, off-label use (20, 23, 96, 97)	Topiramate	Anticonvulsant	Enhanced GABA and reduced AMPA function	12.50–25.00 mg/d	25.00–100.00 mg/d	Weight loss and cognitive impairment, drowsiness, headache, and risk of renal stones

^a^ The recommended dosage is based on age. Patients who are younger than 8 years of age use the minimum therapeutic dose to approximately 1/2 maximum therapeutic dose, such as tiapride (100–350 mg/d). For patients who are older than 8-year-old use the maximum therapeutic dose of 1/2 to maximum therapeutic dose, such as tiapride (350–600 mg/d). ^b^ Transdermal patch. ^C^ TCM, Traditional Chinese Medicine.

### Comorbidity Treatment

#### Comorbid With ADHD (TD+ADHD)

This is one of the most common clinical comorbidities (49). Alpha 2 receptor agonist, such as clonidine and atomoxetine hydrochloride, is the first-line treatment, which has the anti-tic function and improves attention (28, 98). Atomoxetine hydrochloride does not induce or aggravate tics, so it can also be applied to TD children with ADHD (99). Guanfacine is not available in China. There was also successful clinical experience in using methylphenidate for TD+ADHD treatment (21, 100). Central stimulant, mainly methylphenidate, is the second-line treatment of TD+ADHD in China. However, there is a potential risk of aggravating or inducing tics by psychostimulants (21). It is generally advocated that conventional doses of dopamine receptor blockers, such as tiapride, should be combined with low doses of psychostimulants, such as methylphenidate, 1/4~1/2 of the conventional dosage, to treat children with TD+ADHD (101). Such treatment can effectively control the symptoms of ADHD but has little effect on the tic symptoms of most children. Evidence from pharmacological studies conducted over the last decade supports the use of stimulants to prioritize the treatment of debilitating ADHD symptoms in patients with TD/TS.

#### Comorbid With OCD (TD+OCD)

Cognitive-behavioral therapy (CBT) with an exposure/response prevention (ERP) component has the strongest evidence-based effect and is considered to be the first-line treatment for TD+OCD, if available (27, 28). Selective serotonin reuptake inhibitors (SSRIs), such as sertraline, are the first-line pharmacological agents. SSRIs are the only class of medications that has primary efficacy for OCD (27, 28). SSRIs should start with a small dose and gradually increase. Tricyclic antidepressants, such as clomipramine, could be used as a second-line medication for TD+OCD, but with more side effects (59). Newer antidepressants can also be used to treat TD comorbid with OCD. The European clinical guidelines suggest to use risperidone as a first-line choice for TD+OCD (23). Dopamine receptor blockers, such as aripiprazole and risperidone, are often used in combination with SSRIs, such as sertraline, to treat TD with severe OCD symptoms (21, 102).

#### Comorbid With Other Behavioral Disorders

TD cases with other significant behavioral disorders, such as learning difficulties, sleep disorders, self-injurious behaviors, and conduct disorder, should be consulted with or referred to professionals in specialized education, psychological intervention, behavioral therapies, and sleep disorders (103). In some complicated severe cases, it is necessary to timely transfer the patients to advanced pediatric psychiatry and/or neuropsychological services for comprehensive assessment and treatment.

### Refractory TD Treatment

When treatment outcome is not satisfactory as expected, some common scenarios should be investigated to first exclude false refractory TD, such as misdiagnosis, improper medication choice, insufficient dosage, intolerance of side effects, or poor medication compliance. For children with refractory TD/TS in China, it is recommended that such patients should be referred to pediatric psychiatry or multidisciplinary team for evaluation and management. Once the diagnosis of refractory TD is established, a comprehensive treatment plan could include combined medications, newer medications, non-pharmacological therapy, and proper treatment of comorbidities (3, 104).

Some newer medications were reported to be effective for the treatment of adult patients with refractory TD in the Western countries. These include new D1/D5 receptor antagonists (e.g., ecopipam), vesicular monoamine transporter inhibitors (e.g., tetrabenzazine), antagonist of the nicotinic acetylcholine receptors (e.g., mecamylamine), cannabinoids (e.g., tetrahydrocannabinol), glutamatergic blocker (e.g., riluzole), γ-aminobutyric acid (GABA), finasteride, and omega-3 etc (3, 28, 89, 96, 104). However, these newer medications are currently unavailable or not widely used in pediatric clinics in China.

Botulinum toxin injections for the treatment of adolescents and adults with localized and bothersome simple motor tics have been recommended in the Western countries when the benefits of treatment outweigh the risks (3, 27, 28, 96). Various neural regulation therapies have been reported, including repetitive transcranial magnetic stimulation (rTMS), cranial electrotherapy stimulation (CES), and EEG biofeedback. However, the effects of such treatments are non-conclusive, and sometimes controversial (3, 105); therefore, they should be prescribed with caution and reservation. The beneficial effect of deep brain stimulation (DBS) has been more consistently reported in small scale clinical trials for refractory TS (3, 28, 96). But DBS belongs to invasive treatments, so it should only be considered for older children (above 12 years old) or adult refractory TS or special cases, such as the ones with severe self-injurious behaviors. It should also be under multidisciplinary assessments, follow stringent criteria, and ethically approved protocols (3, 26, 28, 78, 96, 105, 106).

### Traditional Chinese Medicine Treatment of TD in China

Pharmacological treatments often have significant side-effects; while currently CBT is not easily assessable in most parts of China. Neither of the non-pharmacological and pharmacological treatments results in complete resolution of tic symptoms. Therefore, a variety of seemingly safer and easier complementary alternative medicine (CAM) have become available to the patients with TD and their caregivers (107).

Traditional Chinese Medicine (TCM), which mainly includes TCM medication and acupuncture that have been developed for the prevention and treatment of various diseases and refined by the Chinese people over thousands of years, is widely available and commonly used all over China. TCM medication could be prescribed as individualized formula, often in the form of liquid decoctions; or in pre-made dry decoctions or granules or tablets, based on some commonly used formula with specific medical ingredients for specific diagnoses or medical purposes (108). Notably, in recent years TCM has drawn increasing attention worldwide, and the ICD-11 will formally include a chapter on TCM classification for the first time in 2022 (109–111).

Based on the TCM diagnostic procedures, patients with TD could be further diagnosed and classified as different specific subtypes of TD, which are defined by different types of yin-yang imbalances of the functional entities of the body and mind (22). In China, TCM could be used to treat TD alone by specialized TCM physicians, often using individualized, freshly made daily liquid decoctions; or used by pediatric neurologists and psychiatrists in combination with Western Medicine, often prescribed as premade formulated granules or tablets (12).

There has been a standardized national guideline for the diagnosis and treatment of TD using TCM in China since 2012, and an updated version in 2019 (22). Some meta-analyses have supported the efficacy and safety of TCM alone and TCM plus Western Medicine in treating patients with TD/TS (112–116). For example, a randomized, placebo-controlled, double-blind clinical study investigated the short-term effectiveness and safety of one premade TCM medication, Ningdong (ND) Granule in pediatric subjects (aged 7–18 years) with TS, showed a 41.39% reduction in the total tic score, while the placebo group showed a 10.79% decrease (117). In another multicenter, double-blind, double-dummy, randomized, placebo-controlled trial, 603 patients with TS aged 5–18 years were randomly assigned to either treatment with placebo (n = 117), or tiapride (n = 123, 200–400 mg/d) or 5-Ling Granule (5-LGr), a proprietary polyherbal product (n = 363, 15.0–22.5 g/d), for 8 weeks; and the results showed that the clinical efficacy of 5-LGr was comparable to tiapride in reducing tics but its safety profile was better than tiapride (118). Changma Xifeng Tablet is another proprietary polyherbal Chinese medicine often used to treat patients with TD in China. In another multicenter, double-blind, double-dummy, randomized, parallel positive drug-controlled trial, patients with TS aged 4–18 years were randomly assigned to treatment with Changma Xifeng Tablets (n = 438) or tiapride (n = 110); and Changma Xifeng Tablet showed similar clinical efficacy as tiapride (86.59% vs. 82.73%) but with fewer side effects as compared with tiapride (0.00% vs. 5.45%) (95). Changma Xifeng Tablet has been approved as one of the first-line TCM for the treatment of pediatric patients with TD by the Chinese National Administration of Traditional Medicine (22). There has also been increasing modern medical research in China to understand the mechanisms of TCM. For example, another commonly used TCM decoction (Xiao-Er-An-Shen) for the treatment of TS in children in mainland China, its beneficial effect was shown to be associated with reversing abnormal changes of neurotransmitter levels and enhancing antioxidant status in an experimental mouse model of TS (119).

Similarly, acupuncture has been demonstrated to be an effective alternative therapy for TD/TS in China (114, 115). Two meta-analyses of seven and ten randomized clinical trials (564 participants and 703 participants, respectively) in China have shown that compared with Western medicine (e.g., haloperidol and risperidone), acupuncture seemed to be more effective in short-term to improve the YGTSS [MD −4.60, 95% CI −5.80 to −3.40) (114) or SMD -0.71, 95%Cl (-1.10, -0.33), Z=3.65, P = 0.0003) (115)]; and the response rate, compared to haloperidol or risperidone [(RR = 1.15, 95% CI (1.05,1.25), Z = 3.05, P = 0.002 (115) or RR = 1.19 (95% CI 1.08 to 1.31, Z = 3.42, P = 0.0006) (114)]. Acupuncture could also be used as an adjuvant therapy to enhance the effect of Western medicine in improving the YGTSS (MD −7.11, 95% CI −8.74 to −5.47) (114).

However, the total number and sample sizes of the reported RCTs on the TCM medications and acupuncture treatment for TD were still relatively small, compared to the RCTs of Western medicine. Therefore, high quality RCTs on TCM treatment of TD/TS remain scarce, and large-scale and well-designed RCTs with rigorous methods of TCM medication and acupuncture for TS are warranted (113).

## Prognosis and Main Determinants of Quality of Life in Patients With TD

The overall prognosis of TD is relatively benign, and most children with TD can grow up to work and live as healthy adults. However, a small fraction of children with TD could carry prolonged tic symptoms and comorbidity into their adulthood that would compromise their quality of life and career.

Nearly half of the pediatric patients with TD would have complete remission in adolescence or adulthood, and about another 30% of them would have alleviated tics in adulthood; up to 20% of the patients with TD would have deferred tics into adulthood or lifelong (43, 120). Only a small fraction (5–10%) of pediatric patients with TD not only experience tic worsening in adulthood but also develop the most severe and debilitating forms of TD, particularly those with comorbidities.

The prognosis of children with TD could be associated with certain risk factors, including a family history of mental or neurological disorders, childhood psychosocial stress, higher childhood tic severity score, smaller caudate volumes, and poor fine-motor control (120, 121). Since TD symptoms can be alleviated or relieved with age and brain development, the prognosis should be deferred until around 18 years old.

In the meantime, comorbid ADHD symptoms tend to decrease in only 20% of children during adolescence but later than tics. The strongest predictor of ADHD in early adulthood is ADHD severity in childhood. Furthermore, having a family history of ADHD or getting special education in childhood also significantly increases the risk of future ADHD. Studies have also shown that comorbid ADHD and OCD tend to persist, with ADHD symptoms (120) and OCD severity in childhood strongly predict OCD in early adulthood. Moreover, the presence of untreated comorbidities could also adversely affect the long-term outcome of patients with TD (120, 121).

TD is a chronic neuropsychiatric disorder that has a significant negative impact on the health-related quality of life (HR-QOL) of patients and their families, if not properly managed. A systematic review (122) and other studies (123–125) indicated that in patients with mild to moderate TS, HR-QOL relates primarily to co-morbidities of ADHD and OCB/OCD. ADHD with predominantly inattentive symptoms, rather than hyperactivity symptoms, was associated with lower QOL (123). However, young patients with severe tics associated with characteristic premonitory urges and a family history of TD appear to be at higher risk for poorer HR-QOL as adults (124). QOL profiles in children reflect more the impact of co-morbid attention-deficit and hyperactivity symptoms, which tend to improve with age, whereas adults’ perception of QOL seems to be more strongly affected by the presence of depression and anxiety symptoms (122). Therefore, early interventions and effectively managing the comorbidities in pediatric patients, as well as proper treatment of depression, anxiety, and other comorbid symptoms in young adult patients with TD, will effectively improve their HR-QOL.

## Summary of TD Diagnosis and Treatment

Clinical diagnosis of TD relies on detailed medical history, careful physical examination, and some auxiliary tests. Direct interview and exam of the children are essential to observe the tics, as well as the general behaviors and mental status, and to identify any additional concomitant sign(s) or symptom(s) to rule out any primary causative medical condition or for differential diagnoses. It needs to keep in mind that tic symptoms can be self-controlled for a short period, so it is easy to overlook and miss the diagnosis at the beginning. At the same time, TD can also be disguised by other prominent symptoms, particularly with comorbidities. Secondary TD, such as tic-like movements caused by rheumatic chorea, epilepsy, and other extrapyramidal disorders, should be excluded. Treatment should be considered at the individual level, gradually apply available non-pharmacological therapy, and pharmacological agents and other interventions with careful measurement of therapeutic effects, side effects, and overall outcomes. A simplified diagnosis and treatment roadmap is shown in **Figure 1**.

## The membership of the Chinese Child Neurology Society Tic Disorders Consortium (grouped by the geographic locations):

1. Beijing Anding Hospital Affiliated to Capital Medical University (Drs. Yan-Jie Qi, Yi Zheng), Beijing, China
2. Peking University First Hospital (Drs. Ying Han, Yu-Wu Jiang, Qin Lin, Zhi-Xian Yang), Beijing, China;
3. Peking University People’s Hospital (Drs. Na Fu, Jiong Qin), Beijing, China;
4. Beijing Children’s Hospital Affiliated to Capital Medical University (Drs. Fang Fang, Yong-Hua Cui), Beijing, China;
5. Children’s Hospital Affiliated to Capital Institute of Pediatrics (Drs. Qian Chen, Li-Wen Wang, Jian Yang), Beijing, China;
6. People’s Liberation Army General Hospital (Drs. Guang Yang, Li-Ping Zou), Beijing, China;
7. Pediatric Hospital Affiliated to Fudan University (Drs. Dao-Kai Sun, Yi Wang, Li-Fei Yu, Shui-Zhen Zhou), Shanghai, China;
8. Shanghai Children’s Medical Center (Drs. Ji-Wen Wang, Zhi-Ping Wang), Shanghai, China;
9. Xinhua Hospital Affiliated to Shanghai Jiaotong Unniversity School of Medicine (Dr. Ling Li), Shanghai, China;
10. Children’s Hospital Affiliated to Chongqing Medical University (Drs. Fang-Cheng Cai, Si-Qi Hong, Li Jiang), Chongqing, China;
11. Tianjin Children’s Hospital (Dr. Yu-Qin Zhang), Tianjin, China;
12. Wuhan Children’s Hospital, Tongji Medical College, Huazhong University of Science and Technology (Drs. Zhi-Sheng Liu, Qing Lu, Dan Sun), Wuhan, Hubei Province, China;
13. Tongji Hospital of Tongji Medical College, Huazhong University of Science and Technology (Dr. Yan Liu), Wuhan, Hubei Province, China;
14. Xiangya Hospital of Central South University (Drs. Jing Peng, Fei Yin), Changsha, Hunan Province, China;
15. Cangzhou People’s Hospital of Hebei Province (Dr. Rong Wang), Cangzhou, Hebei Province, China;
16. Children’s Hospital of Hebei Province (Dr. Rong-Fu Shi), Shijiazhuang, Hebei Province, China;
17. Xinxiang Medical College (Dr. Xue-Peng Guo), Xinxiang, Henan Province, China;
18. The Third Affiliated Hospital of Xinxiang Medical College (Dr. Jia-Qin Wang), Xinxiang, Henan Province, China;
19. People’s Hospital of Henan Province (Dr. Li Gao), Zhengzhou, Henan Province, China;
20. The First Affiliated Hospital of Anhui Medical University (Drs. Jiu-Lai Tang, De Wu), Hefei, Anhui Province, China;
21. People’s Hospital of Hainan Province (Dr. Li-Shuang Que), Haikou, Hainan Province, China;
22. Union Hospital Affiliated to Fujian Medical University (Drs. Yan-Hui Chen, Jun Hu), Fuzhou, Fujian Province, China;
23. The First Affiliated Hospital of Guangxi Medical University (Dr. Yun-Li Han), Nanning, Guangxi Province, China;
24. Guangzhou Medical Center for Women and Children (Drs. Jian-Ning Mai, Si-Da Yang), Guangzhou, Guangdong Province, China;
25. Shenzhen Children’s Hospital (Drs. Bing-Rang Yang, Yan Hu, Jian-Xiang Liao), Shenzhen, Guangdong Province, China;
26. Haerbin Children’s Hospital (Drs. Chun-Yu Wang, Wei Wang), Haerbin, Heilongjiang Province;
27. The First Affiliated Hospital of Jilin University (Drs. Dong Liang, Jian-Min Liang), Changchun, Jilin Province, China;
28. Children’s Hospital of Jiangxi Province (Dr. Jian-Min Zhong), Nanchang, Jiangxi Province, China;
29. The Second Hospital Affiliated to Lanzhou University (Dr. Yong-Qian Chen), Lanzhou, Gansu Province, China;
30. General Hospital of Ningxia Medical University (Dr. Guang-Bo Bian), Yinchuan, Ningxia, China;
31. Qinghai Maternal and Child Health Care Hospital (Dr. Shou-Lei Wang), Xining, Qinghai Province, China;
32. Qilu Hospital of Shandong University (Drs. Bao-Min Li, Ruo-Peng Sun), Jinan, Shandong Province, China;
33. Children’s Hospital Affiliated to Suzhou University (Dr. Yan Li), Suzhou, Jiangsu Province, China;
34. The Second Affiliated Hospital of Xi’an Jiaotong University Medical College (Drs. Shao-Ping Huang, Lin Yang), Xi’an, Shaanxi Province, China;
35. Children’s Hospital of Shanxi Province (Dr. Hong Han), Taiyuan, Shanxi Province, China;
36. Shengjing Hospital Affiliated to Chinese Medical Sciences University (Drs. Hua Wang, Jun-Mei Zhang), Shenyang, Liaoning Province, China;
37. Children’s Hospital Affiliated to Zhejiang University (Dr. Feng Gao), Hangzhou, Zhejiang, China;
38. Hangzhou Children’s Hospital (Dr. Guang-Qian Li), Hangzhou, Zhejiang Province, China;
39. The First People’s Hospital of Yunnan (Dr. Chun-Hui Tang), Kunming, Yunnan Province, China;
40. Huaxi Second Hospital of Sichuan University (Drs. Rong Luo, You-Quan Zhong), Chengdu, Sichuan Province, China;
41. Affiliated Hospital of Zunyi Medical College (Dr. Xiao-Mei Shu), Zunyi, Guizhou Province, China.
42. Macao Earl Count General Hospital (Dr. Xiang Cai), Macao Macao, China;
43. The Affiliated Hospital of Inner Mongolia Medical University (Dr. Guang-Lu Yang), Hohhot, Inner Mongolia Autonomous Region, China;
44. Wulumuqi Children’s Hospital (Dr. Xi Chen), Urumqi, Xinjiang Autonomous Region, China;
45. The Xinjiang Uygur Autonomous Region people’s Hospital (Dr. Yan Sun), Urumqi, Xinjiang Autonomous Region, China;
46. People’s Hospital of Tibet Autonomous Region (Dr. Rong Zhao), Lhasa, Tibet Autonomous Region, China;
47. Montreal Neurological Institute and Hospital, McGill University (Dr. Lan Xiong), Montreal, Quebec Province, Canada;

## Author Contributions

All authors listed have made substantial, direct, and intellectual contribution to the work and approved it for publication.

## Conflict of Interest

The authors declare that the research was conducted in the absence of any commercial or financial relationships that could be construed as a potential conflict of interest.
