# Supplementary material for: Current Status, Diagnosis, and Treatment Recommendation for Tic Disorders in China
Source: Front Psychiatry. 2020 Aug 13;11:774. doi: 10.3389/fpsyt.2020.00774 (PMC7438753; doi:10.3389/fpsyt.2020.00774)
Supplement: Supplementary file 1 [file Table_1.docx]

**Table 1. Manifestation and classification of tics**

| Tic Type | Simple Tic | Complex Tic |
| --- | --- | --- |
| Motor Tic | Blink of eye/oblique eye, frown, eyebrows, open mouth, loll tongue, tapir mouth/crooked mouth, lick lips, crumpled nose, nod /raise/shake/swivel head, torticollis, shrug shoulders, move fingers/toes, rub hands, clench fist, move wrists, lift/stretch/internal rotate arms, stretch/shake legs, step/pedal foot, extend/bend knees, extend/bend coxa, lift chest, hold abdomen, twist waists and so on. | Lift eyebrows and wink, make faces, eyeball rotate, knob fingers, swing/clap hands, wave arms, stab action, flick limbs, hit chest with fists, bend waist, mandible touch the knee, twist trunk, move up and down, squat, kneel posture, kick legs, knee joint, stamp foot, jump, hop, throw, beat, touch, sniff, touch the hair, walk in circles, walk backwards and so on. |
| Vocal Tic | Single-tone, sniffing, clearing throat, roaring, humming, coughing, squeaky sound, screaming, shouting, grunting, spitting, whistling, sucking, barking, tweeting and so on. | Single word/phrase/clause/sentence, repeat single word or phrase, repeat sentence, imitate speech, obscene language and so on. |
