# Supplementary material for: Current Status, Diagnosis, and Treatment Recommendation for Tic Disorders in China
Source: Front Psychiatry. 2020 Aug 13;11:774. doi: 10.3389/fpsyt.2020.00774 (PMC7438753; doi:10.3389/fpsyt.2020.00774)
Supplement: Supplementary file 2 [file Table_2.docx]

**Table 2. Recommended medications in the treatment of TD**

| **Recommendation**  **References** | **Drug Name** | **Type** | **Mechanism of Action** | **Initial Dose** | **Therapeutic Dose ^a^** | **Common Side Effects** |
| --- | --- | --- | --- | --- | --- | --- |
| First-line Med  (20, 91, 105) | Tiapride | Antipsychotic, typical neuroleptic | D2 receptor blockade | 50-100 mg/d | 100-600 mg/d | Somnolence, gastrointestinal reactions |
| First-line Med  (20, 21, 78, 80, 91, 105, 106) | Aripiprazole | Antipsychotic, atypical neuroleptic | Partial agonist of dopaminergic (D2, D3, and D4 receptor) and serotonergic (5-HT1A and 5-HT2C) receptors | 1.25-5.00 mg/d | 2.50-20.00 mg/d | Somnolence, weight gain, gastrointestinal reactions |
| First-line Med (TD+ADHD)  (20, 21, 107-109) | Clonidine ^b^ | Alpha agonist | α2 adrenergic receptor agonist | 1.0 mg/w | 1.0-2.0 mg/w | Somnolence, dry mouth, dizziness, headache, fatigue, occasional orthostatic hypotension, and bradycardia |
| First-line Med  (81, 99) | Changma Xifeng Tables | TCM ^c^ | Unknown | 0.53-1.59 g/d | 1.59-4.77 g/d | No obvious adverse reaction |
| First-line Med  (81, 110) | Jiuwei Xifeng Granule | TCM | Unknown | 6.0-12.0 g/d | 12.0-24.0 g/d | No obvious adverse reaction |
| Second-line drug  (20, 21, 91, 105) | Haloperidol | Antipsychotic, typical neuroleptic | D2 receptor blockade | 0.25-1.00 mg/d | 1.00-6.00 mg/d | Somnolence, extrapyramidal symptoms, increased appetite, and hepatic insufficiency |
| Second-line drug, off-label use  (20, 21, 91, 105) | Risperidone | Antipsychotic, atypical neuroleptic | 5-HT2 receptor antagonist at low doses andD2 antagonist at high doses | 0.25-1.00 mg/d | 1.00-4.00 mg/d | Weight gain and extrapyramidal response |
| Second-line drug, off-label use  (20, 21, 82, 111) | Topiramate | Anticonvulsant | Enhanced GABA and reduced AMPA function | 12.50-25.00 mg/d | 25.00-100.00 mg/d | Weight loss and cognitive impairment, drowsiness, headache, and risk of renal stones |

Notes: ^a^ The recommended dosage is based on age. Patients who are younger than 8 years of age use the minimum therapeutic dose to approximately 1/2 maximum therapeutic dose, such as tiapride (100-350mg/d). For patients who are older than 8-year-old use the maximum therapeutic dose of 1/2 to maximum therapeutic dose, such as tiapride (350-600mg/d). ^b^ Transdermal patch. ^C^ TCM: Traditional Chinese Medicine.
